# One-pot synthesis of graphene hydrogel-anchored cobalt-copper nanoparticles and their catalysis in hydrogen generation from ammonia borane

**DOI:** 10.3906/kim-2107-32

**Published:** 2021-09-12

**Authors:** Ibtihel ZAIER, Önder METİN

**Affiliations:** 1 Department of Nanoscience and Nanoengineering, Division of Nanomaterials, Atatürk University, Erzurum, Turkey; 2 Department of Chemistry, College of Sciences, Koç University, İstanbul, Turkey

**Keywords:** Graphene hydrogel, cobalt-copper, nanoparticles, nanocatalyst, ammonia borane, hydrogen generation

## Abstract

We reported a facile one-pot synthesis of bimetallic CoCu nanoparticles (NPs) anchored on graphene hydrogel (GH-CoCu) as catalysts in hydrogen generation from the hydrolysis of ammonia borane (HAB). The presented novel one-pot method composed of the reduction of the mixture of graphene oxide, cobalt(II), and copper(II) acetate tetrahydrates by aqueous ethylene glycol solution in a teflon-coated stainless-steel reactor at 180 °C. The structure of the yielded GH-CoCu nanocatalysts was characterized by TEM, SEM, XRD, XPS, and ICP-MS. This is the first example of both the synthesis of bimetallic CoCu NPs anchored on GH and the testing of a hydrothermally prepared noble metal-free GH-bimetallic nanocomposites as catalysts for the HAB. The presented in situ synthesis protocol allowed us to prepare different metal compositions and investigating their catalysis in the AB hydrolysis, where the best catalytic activity was accomplished by the GH-Co_33_Cu_67_ nanocatalysts. The obtained GH-CoCu nanocatalysts exhibited a remarkable catalytic performance in the HAB by providing the highest hydrogen generation rate of 1015.809 ml H_2_ g_catalyst_^−1^ min^−1^ at room temperature. This study has a potential to pave a way for the development of other GH-based bimetallic nanocatalysts that could be used in different applications.

## 1. Introduction

One of the current global concerns is focused on how to manage the continuously growing energy demand with the existing fossil fuel supplies, which are likely to face shortages in the future as well as they are pointed as the origin of the environmental problems. Therefore, the search for alternative energy sources that might be utilized all over the world regardless of the geographic location has been an attractive research topic. In this regard, hydrogen has been regarded as the best candidate because it is a clean and efficient energy carrier and generated from numerous sources including water [1]. Moreover, it offers the possibility of overcoming obstacles encountered with the fossil fuels and renewable energy sources [2, 3]. The studies on the utilization of hydrogen as an energy carrier in daily life applications ranging from personal electronics to the large grid systems are proceeded under the so-called “*Hydrogen Economy*” title [4, 5]. The ongoing key challenge in the *Hydrogen Economy* is the lack of practical, safe, and economical systems or materials for hydrogen storage [6]. Among the established systems and materials, chemical hydrogen storage materials possessing high gravimetric/volumetric hydrogen density are deemed as one of the most favorable solutions to the hydrogen storage problem [7, 8]. In this context, B-N based hydrides, namely amine-boranes, have high potential to be used as hydrogen storage materials owing to their high hydrogen densities [9–11]. Among amine boranes, ammonia borane (AB) was particularly the object of many studies and reported as a promising hydrogen storage material owing to its impressive gravimetric hydrogen content of 19.6 wt%, stability under ambient conditions, and nontoxicity [12, 13]. There are three main routes to release the hydrogen stored in AB, namely thermal decomposition, the catalytic dehydrogenation in organic solvents (dehydrocoupling), and the catalytic solvolysis [14]. Considering the mobile applications, the last method is the most convenient one for hydrogen generation from AB since it does not require high temperature for releasing 3 equivalence hydrogen at ambient temperature, even at low temperatures [12, 15, 16]. The reaction equation for the AB hydrolysis can be expressed as the following (Eqn. 1) [17]:


(1) $${\text{H}}_{3}{\text{NBH}}_{3}\;(\text{aq})+2\;{\text{H}}_{2}\text{O }(\text{l})\overset{\text{catalyst}}{\to }\;({\text{NH}}_{4}){\text{BO}}_{2}\;(\text{aq})+3{\text{H}}_{2}\;(\text{g})$$

Precious metal nanoparticles (NPs), particularly Ru, Rh, Pd, and Pt, supported on Al_2_O_3_ [15], SiO_2_ [15, 18, 19], graphene [20, 21], graphitic carbon nitride [22, 23], and other support materials [24–27] were the most efficient catalysts for the HAB. To decrease the cost of these catalysts yet maintaining their catalytic activity, the bimetallic nanocatalysts composed of nonnoble metals (i.e., Fe, Co, Ni, and Cu) and noble metals have been reported to be highly efficient catalysts in hydrogen generation from the HAB [28–30]. However, the ultimate target for the practical applications is the development of efficient noble metal-free nanocatalysts for the AB hydrolysis [31–33]. Among the nonnoble metal catalysts, Co nanocatalysts have received much attention due to their high catalytic activity compared to other nonnoble metals [34–40]. Co NPs anchored on various support materials including Al_2_O_3_ [31], PAMAM [41], and zeolite [28] were reported to enhance the catalytic activity and stability of in the HAB. Additionally, graphene-based support materials have been used for the further performance enhancement of Co NPs compared to the other type of supports thanks to their unique two-dimensional structure and favorable electronic properties [42–44]. However, Co is one of the most expensive metal among the nonnoble metals. Therefore, to further improve the catalytic activity of Co NPs by lowering its cost in the AB hydrolysis, bimetallic CoM NPs, where M is the first-row transition metal, were investigated to take the advantage of the synergetic effects aroused between the second metal and cobalt. Although copper is generally reported to be shown low catalytic activity in the AB hydrolysis [45–48], bimetallic CoCu nanocatalysts showed enhanced catalytic activity compared to their monometallic counterparts [49–52]. Additionally, it was reported that the use of graphene-based support materials improves the catalytic activity of CoCu nanocatalysts in the AB hydrolysis [53–56]. However, when these CoCu-based nanocatalyst systems were examined, it could be seen that they were mostly prepared by using two-step protocol involving the impregnation of metal precursors into the support material followed by chemical reduction using mostly NaBH_4_ as the reducing agent. In this regard, there is no example of a one-pot synthesis of bimetallic CoCu nanocatalysts anchored on a support material in which both support material and metal NPs are generated simultaneously.

In this study, we report a novel one-pot synthesis of bimetallic CoCu NPs anchored on graphene hydrogel (GH-CoCu) via a surfactant-free hydrothermal method. The idea was to evaluate the combination of graphene properties and the synergy between Co-Cu metals to achieve a cost-effective yet efficient nanocatalyst for the AB hydrolysis. The procedure was based on the concurrent self-assembly of graphene oxide (GO) into GH and the reduction of Co(II) and Cu(II) ions via a one-pot hydrothermal synthesis conducted at mild conditions. The yielded GH-CoCu nanocatalysts were characterized by TEM, SEM-EDS, XRD, XPS, and ICP-MS analysis before their catalytic tests in the AB hydrolysis. The GH-CoCu nanocatalysts provided a relatively high catalytic activity with the highest hydrogen generation rate of 1015.809 mL H_2_ g_catalyst_^−1^ min^−1^, which is higher than those of their monometallic counterparts.

## 2. Materials and methods

### 2.1. Chemicals

All the chemicals used for the synthesis of graphene oxide, graphene hydrogel, GH-based nanocatalysts and catalytic studies were purchased from Merck and used as received. Deionized water purified by Milli-Q System was used in all experiments.

### 2.2. Instrumentation

Transmission electron microscope (TEM) images were recorded on a Hitachi HT7800 TEM instrument operating at 120 kV and FEI TALOS F200S instrument operating at 200 kV XPS; analysis was performed on a Thermo K-Alpha X-ray photoelectron spectrometer (XPS) equipped with Al-Kα source. XRD diffractograms of the prepared nanocomposites were acquired by using a BRUKER D8 DISCOVER XRD instrument equipped with a 1D fast detector. The acceleration voltage was 40 V, and the samples were examined at working distance of 55 mm in the 2θ range of 10–90θ. The metal contents of the synthesized nanocatalysts were determined by the ICP-MS (Inductively Coupled Plasma Mass Spectroscopy, Agilent 7800) after the complete dissolution of the sample in a mixture of HNO_3_/HCl (1/3 ratio). SEM images were recorded on a FEI Nova Nano SEM 450 instrument.

### 2.3. One-pot synthesis of graphene hydrogel (GH) and GH-CoCu nanocatalysts

Bare graphene hydrogel (GH) and GH-CoCu nanocatalysts were prepared by the hydrothermal reduction of graphene oxide (GO) and metal precursors in aqueous ethylene glycol (EG) solution [57]. GO was synthesized by using modified Hummer’s method [58, 59]. The details of the GO synthesis as well as its detailed structural characterization were reported in our previous publications [48][60].

In a typical procedure for the synthesis of GH-CoCu nanocatalysts, 200 mg of as-prepared dry GO was sonicated in 15 mL of water for 1 h in order to achieve a clear brown solution. Afterwards, 43.1 mg of Co(CH_3_COO)_2_.4H_2_O and 31.4 mg of Cu(CH_3_COO)_2_.4H_2_O were dissolved in 55 mL of EG, and the resultant solution was transferred into the aqueous GO suspension. Next, pH value of the mixture was adjusted to 10 by using a 5M NaOH solution. Finally, the mixture was transferred to a Teflon-lined stainless-steel autoclave (100 mL) followed by a hydrothermal treatment at 180 °C for 8 h. The obtained nanocomposites were filtrated, then washed with water and ethanol. The ethanol was evaporated using a rotary evaporator and the nanocomposites were dried at 80 °C for 24 h. Three compositions were considered for the synthesis of GH-CoCu nanocomposites based on the percentage of metal precursors (expressed as molar%): GH-Co_62.5_ Cu_37.5_, GH-Co_50_Cu_50_ and GH-Co_37.5_ Cu_62.5_. GH-Co and GH-Cu were also prepared by following the same procedure to investigate their catalytic performances in comparison with their bimetallic counterpart. To investigate the effect of the change of crystallinity and microstructure on the catalytic performance, as-prepared GH-CoCu nanocatalysts were annealed at 400 °C for 1 h under Ar/H_2_ flow.

### 2.4. GH-CoCu catalyzed HAB

The catalytic activity of as-prepared nanocatalysts in the AB hydrolysis was determined by measuring the rate of hydrogen generation via water-burette system [33, 61, 62]. In a typical catalytic hydrolysis reaction, 10 mg of the prepared nanocatalysts were dispersed in 7.0 mL of water and placed in the hydrolysis reactor. Next, 1.0 mL of aqueous NaBH_4_ (SB) solution was injected to reduce the metal ions. After the completion of the NPs formation and the hydrogen generation from the NaBH_4_ stopped, 2.0 mL of AB solution was injected into the aqueous mixture under vigorous stirring, and hydrogen gas evolution was measured every certain time intervals. The catalytic AB hydrolysis using the annealed nanocatalyst was tested by using the same protocol, except the reduction with NaBH_4_.

## 3. Results and discussion

### 3.1. One-pot synthesis of graphene hydrogel supported cobalt-copper nanoparticles

**Figure 1:**
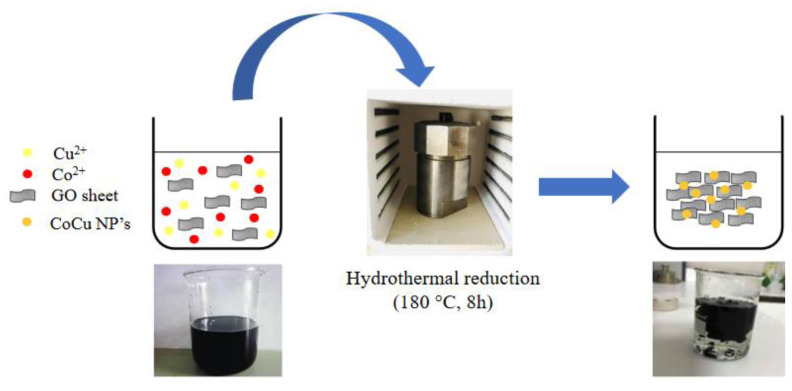
A schematic representation of the synthesis of the GH-CoCu nanocomposites by the simultaneous hydrothermal reduction of GO and Co^2+^/Cu^2+^ ions in the EG-water (55:15) mixture at pH=10.

The presented one-pot protocol for the synthesis of GH-CoCu nanocomposites included two simultaneous processes, namely the reduction and the self-assembly of GO layers into the GH and the reduction of metal precursors into the metallic NPs supported on the formed GH network (Figure 1). However, many simultaneous reactions, including direct decarboxylation, epoxide ring opening-rearrangement-decarboxylation, and C-C cleavage-decarboxylation occur during the hydrothermal reduction of GO [63]. The GO layers went through a self-assembly process by many π-π interactions and van der Waals forces to form a graphene hydrogel network [64]. In the presented protocol, the second process was the reduction of metal ions in the aqueous EG solution, where the yielded GH network served both as surfactant and support material for the stabilization of in situ generated metal NPs. The OH groups present on EG provided it with reducing and coordinating ability, which allowed the synthesis of CoCu NPs by controlling their size and shape. The coordinating properties (i) makes EG a water-equivalent solvent in which metal-salt precursors can be dissolved properly, (ii) controls the nucleation, growth, and agglomeration of particles, since EG, like all polyols, can be adsorbed on the particle surface and provides excellent stabilization of the colloidal NPs [65]. EG has a weak reducing ability at room temperature, but processing at temperatures around its boiling point boosts its reducing power. The presented protocol combining the hydrothermal and polyol method made the reduction of Co(II) and Cu(II) ions possible at a relatively low temperature, which is even lower than the boiling temperature of EG (197 °C). However, it should be noted that temperature was a key factor affecting both the formation of GH network and the CoCu bimetallic NPs. For example, it was found that performing the synthesis at 180 °C was not only favorable for the self-assembly of GO layers into GH [63, 66, 67] but also a suitable temperature for reaching the boiling point of EG without further concern about the applied pressure inside the autoclave. Unlike Ni(II) and Cu(II) ions, the reduction of Co(II) ions with a polyol process does not require alkaline medium particularly when Co(II) acetate is used as a precursor because the basicity of the acetate ion is sufficient to deprotonate a EG molecule [65]. It was noticed that the pH value of the reaction medium after the hydrothermal treatment was extremely acidic (pH= 2.1–2.8), which was attributed to the production of CO_2_ during the gelation of GO [63]. High amount of non-reduced metal ions was found in the reaction solution after the hydrothermal synthesis since the reduced metal cations deposited on GH network leached out into solution due to the low pH value. Therefore, we thought that the adjustment of pH value to the basic medium was necessary for the successful synthesis of GH-CoCu nanocomposites. However, after the pH value adjustment, the yielded GH-CoCu nanocomposites did not possess a thin and firm cylindrical shape (Figure 1) like those achieved previously by the same method for the synthesis of GH-Pd nanocomposites without pH value adjustment [57]. We obtained thicker and easily breakable cylinders which was attributed to the alkaline medium reported to promote the electrostatic repulsions responsible for the formation of less compact graphene hydrogels [68].

### 3.2. The characterization of the GH-CoCu nanocatalysts

**Figure 2:**
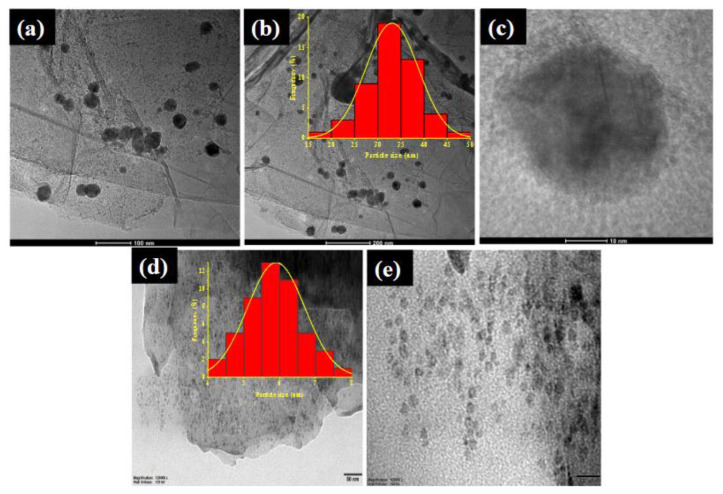
TEM images of the GH-CoCu nanocatalysts before (**a,b,c**) and after annealing at 400 °C for 1h under Ar/H_2_ flow (**d,e**).

Figure 2 shows the representative TEM images of the GH-CoCu nanocatalysts before and after the annealing under Ar/H_2_ at 400 °C. As it can be concluded from the TEM images, before the annealing procedure, as-synthesized CoCu NPs have mostly spherical shape with an average particle size of 33 nm dispersed on the GH nanosheets (Figure 2a–2c). However, the annealed GH-CoCu nanocomposites exhibited a more homogeneous particle size distribution with less agglomeration and smaller particle size (5–6 nm) after the annealing process (Figure 2d–e), which might be attributed to the incomplete reduction of the metal precursors before the annealing [69, 70].

In order to determine the experimental alloy compositions, the real molar ratio of Co-Cu metals in the synthesized nanocatalysts was determined by performing a series of ICP-MS analysis on them, and the obtained results were depicted in Table. As it can be concluded from the Table, there was a difference between the theoretical (based on the initial metal salt amount) and the experimental (determined by the ICP-MS analysis), which was most probably due to the difference in the reduction behavior of the metal precursors in the presented synthesis conditions. Additionally, it might also be attributed to alloying of Co-Cu only at certain compositions. It should also be noted that annealing procedure did not change the composition at all. As a result of the ICP-MS studies, GH-CoCu nanocatalysts with three different alloy compositions, namely Co_33_Cu_67_, Co_45_Cu_55_, and Co_23_Cu_77_, were synthesized and tested as catalysts in the HAB.

**Figure 3:**
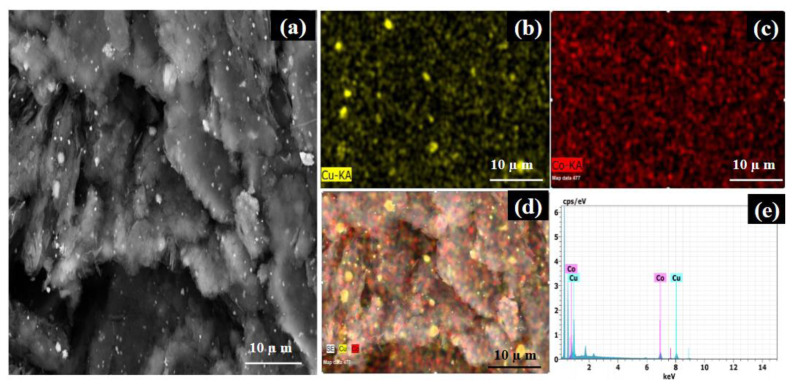
a) SEM, (b–d) SEM-EDS elemental mapping images, (e) SEM-EDS spectrum of the GH-Co_33_Cu_67_ nanocatalysts.

To show the distribution of Co and Cu elements in the GH-CoCu nanocatalyst structure along with proving the alloy formation between Co and Cu metals, we recorded the SEM image and the associated EDS elemental mapping images of the nanocatalyst (Figure 3). A representative SEM image of the GH-Co_33_Cu_67_ nanocatalysts displayed the formation of the NPs possessing diverse particle sizes deposited on GH networks (Figure 3a). The SEM-EDS elemental images for Co (Figure 3b) and Cu (Figure 3c) indicated the good distribution of Co and Cu elements over the GH networks. Moreover, the overlapped elemental mapping image of Co and Cu elements (Figure 3d) confirmed the formation of CoCu alloy NPs in the nanocatalyst structure. Based on the SEM-EDS spectra given in Figure 3e, the Co/Cu atomic ratio was found to be very close to 1.0, which was in consistent with the theoretical ratio calculated by using the metal precursors amount used in the synthesis.

**Figure 4:**
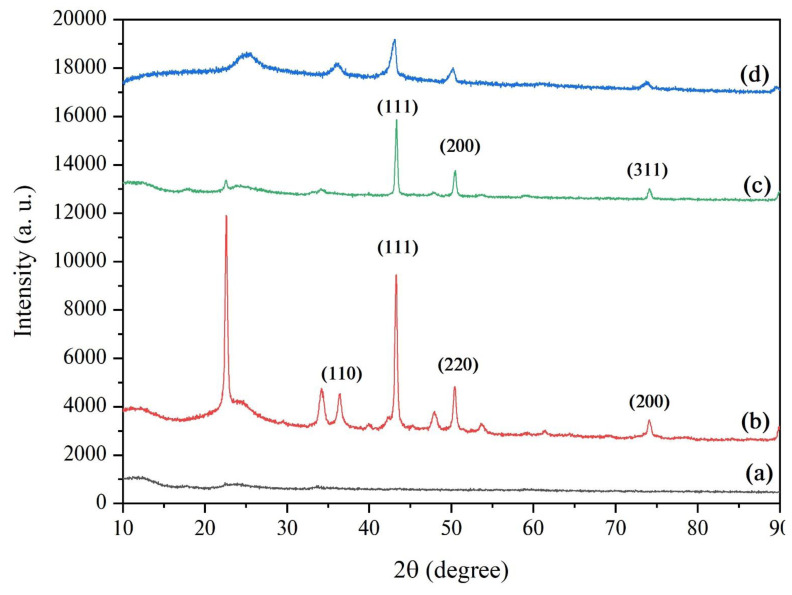
XRD patterns of a) GH-Co, b) GH-Cu, c) GH-Co_33_Cu_67_ and d) annealed GH-Co_33_Cu_67_.

Powder XRD analysis of the as-synthesized GH-Co_33_Cu_67_ nanocatalysts was carried out to obtain information about the crystal structure of the generated CoCu NPs and to further investigate the alloy formation. Figure 4 shows the XRD patterns of the prepared GH-Co_33_Cu_67_ nanocatalysts and their monometallic counterparts. The observation of sharp peaks in all XRD patterns indicated the highly crystalline materials with relatively large particle size except for the monometallic counterpart GH-Co for which the XRD pattern (Figure 4a) showed no obvious peaks revealing the low crystallinity or amorphous structure of the yielded nanocomposite. The peak observed at 2θ = 23.5° corresponds to the (002) plane of carbon network (JCPDS 26-1076), and it is attributed to the conjugated system resulting from the reduction of GO layers upon hydrothermal process [71, 72]. The XRD pattern of the GH-Cu nanocatalysts showed the characteristic peaks of both fcc metallic Cu and copper oxide, indicating the oxidation of Cu during the synthesis (Figure 4b). In the case of XRD pattern of GH-Co_33_Cu_67_ nanocatalyst (Figure 4c), there were only three main peaks observable at 2θ= 43.39, 50.46, and 74.19°, which were attributed to (111), (200), and (311) planes of the fcc CoCu phase, indicating the solid-solution formation (alloy) between the Co and Cu atoms. These results are in line with the data obtained from SEM-EDS analysis. The annealed GH-Co_33_Cu_67_ nanocatalysts exhibited a very similar XRD pattern to the non-annealed one (Figure 4d).

**Figure 5:**
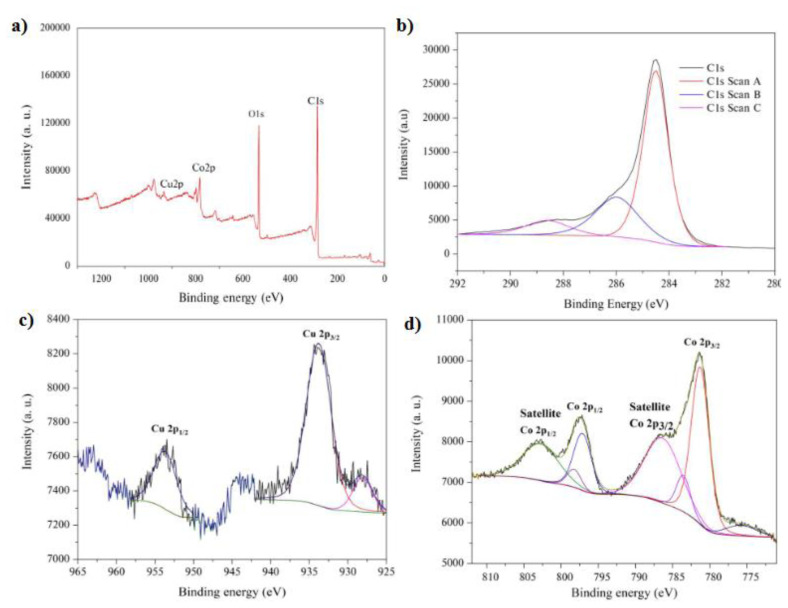
XPS survey scan spectrum **a)**, the deconvoluted high-resolution XPS spectra of **b)** C 1s, **c)** Cu 2p, and **d)** Co 2p core-levels for the GH-Co_33_Cu_67_ nanocatalysts.

The oxidation states of Cu and Co atoms on the surface of GH-Co_33_Cu_67_ nanocatalysts were analyzed by XPS analysis. The XPS survey spectrum of the synthesized GH-Co_33_Cu_67_ nanocatalyst (Figure 5a) confirmed the existence of C, O, Co, and Cu atoms in the structure. The XPS spectrum for C 1s (Figure 5b) shows almost no peaks of carbons binding to oxygen except the small peak centered at 285.98 eV which reveals the reduction of the majority of oxygen-containing functional groups after the hydrothermal treatment. The C-C and π-π* peaks situated at 284.41 and 288.55 eV, respectively, indicate the restoration of sp_2_ carbon network in the formed graphene hydrogel [73]. Figure 5c shows the deconvoluted XPS spectrum for the Cu2p region in which two major peaks along with two minor ones with assymetric shapes were observable. The major peaks located at binding energies (BEs) of 933.2 and 952.9 eV with spin-orbit components (Δ_metal_=19.7 eV) were attributed to the Cu2p_3/2_ and Cu2p_1/2_ core-levels of metallic Cu, while other minor ones indicated the presence of copper oxide species, most probably Cu_2_O [74, 75]. It should be noted that there is a slight shift (≈ 0.2 eV) observable on both peaks indicating the electron transfer from Cu species to Co. Figure 5d shows the deconvulated XPS spectrum Co2p core-level of GH-Co_33_Cu_67_ nanocatalysts in which there were two major peaks observed at BEs of 780.5 and 797.5 eV along with two satellite features at BEs of 786.48 and 802.98 eV that were assigned to Co2p_3/2_ and Co2p_1/2_ peaks of surface oxidized Co species most probably due to exposure to air during the sample preparation for XPS analysis [74].

### 3.3. GH-CoCu catalyzed HAB and the kinetic study

**Figure 6:**
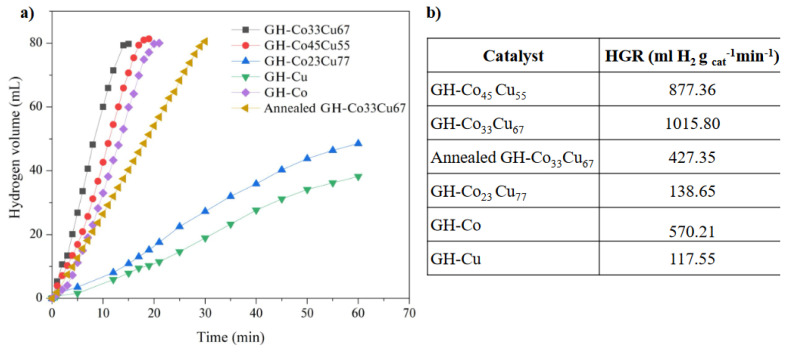
**a)** The volume of hydrogen versus time plots for the HAB catalyzed by GH-CoCu nanocatalysts with different compositions at 25 ± 0.5°C, **b)** the hydrogen generation rates (HGR) calculated for all the tested catalysts.

The catalytic activity of GH-CoCu nanocatalysts was investigated in the HAB via monitoring the hydrogen evolution versus time. Firstly, it is noteworthy to mention that as-prepared GH-CoCu nanocatalysts provided a low catalytic activity in the HAB when they were used directly without any treatment. In this regard, it was thought that the presence of water in the hydrothermal reduction process and the washing/cleaning procedures after the hydrothermal synthesis promoted the surface oxidation of the CoCu NPs on GH nanosheets, and it is well-known that nanoscale Co and Cu metals are highly sensitive to oxidation upon their exposure to air and water. Hence, to eliminate the surface oxides from CoCu NPs, we used a tiny amount of NaBH_4_ reduction before starting the catalytic HAB studies. A detailed kinetic study was performed on the catalytic HAB the catalyst amount, AB amount, and temperature. Before the kinetic studies, the effect of CoCu alloy composition on the catalytic activity of GH-CoCu nanocatalysts in the HAB was investigated by studying the catalytic activity of three different CoCu compositions as well as the monometallic counterparts. As clearly be concluded by the examination of Figure 6a, all tested alloy compositions of GH-CoCu nanocatalysts showed higher catalytic activity compared to the monometallic counterparts in terms of the hydrogen generation rate (HGR). Among the tested CoCu alloy compositions, GH-Co_33_Cu_67_ nanocatalysts provided the highest hydrogen generation rate (1015.809 ml H_2_.g_catalyst_^−1^.min^−1^). Additionally, it should be noted that the catalytic activity of the GH-Co_33_Cu_67_ nanocatalysts dropped after the annealing process. Consequently, the non-annealed GH-Co_33_Cu_67_ nanocatalysts were used for the kinetic studies.

The hydrogen generation rates (HGR) of all tested catalysts are presented in Figure 6b. The HGR of the bimetallic GH-Co_33_Cu_67_ reached 1015.80 mL H_2_ g_catalyst_^−1^ min^−1^, which was much higher than the those provided by GH-Co (570.21 ml H_2_ g_catalyst_^−1^ min^−1^) and GH-Cu (117.55 ml H_2_ g_catalyst_^−1^ min^−1^). These results are clear evidence for proving the alloy formation between Co-Cu metals and the synergistic effects that were aroused between them. The table depicted in Figure 6b shows the catalytic activities of CoCu-based catalysts that have been tested in AB hydrolysis, among which our GH-Co_33_Cu_67_ nanocatalysts have the highest catalytic activity in terms of the HGR values. It is evident that the synthesis method, the operational conditions, and the support nature have a deep impact on the catalyst performance. Considering the advantages of the presented one-pot synthesis protocol for the synthesis of GH-CoCu nanocatalysts, which is the first example in this manner, and their high catalytic activity in the HAB, the current study has the potential to open a new way for the employment of GH-CoCu nanocatalysts in different applications.

**Figure 7:**
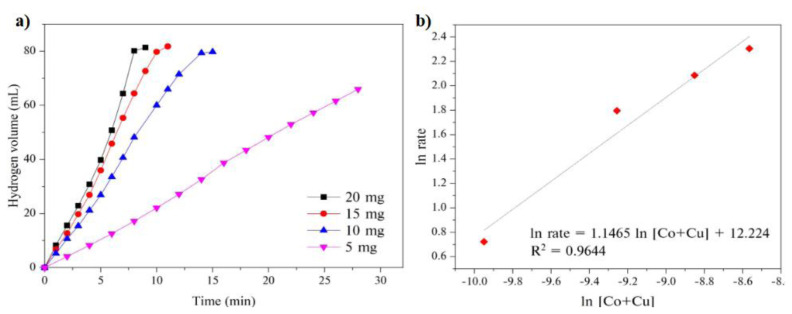
**a)** The plots of the volume of hydrogen versus time for AB hydrolysis catalyzed by GH-Co_33_Cu_67_ with different catalyst concentrations at 25 °C. **b)** the plots of hydrogen generation rate versus concentration of GH-Co_33_Cu_67_, both in logarithmic scale.

After finding the most active composition as GH-Co_33_Cu_67_ in the HAB, the effect of catalyst concentrations on its activity in the AB hydrolysis was investigated by performing the catalytic hydrolysis reaction with different catalyst amounts in the range of 5–20 mg, which corresponds to a metal molar range from 4.76 to 19.05 10^−2^ mM. The amount of AB was kept at 1.0 mmol during these experiments. Figure 7a shows the plots of the volume of hydrogen versus time for the AB hydrolysis catalyzed by different GH-Co_33_Cu_67_ amounts. As expected, the reaction time for the complete hydrogen generation from the 1 mmol of AB decreased, while the hydrogen generation rate increased with increasing catalyst amount. The hydrogen generation rate calculated from the linear portion of each plot was plotted against the catalyst concentration, both in logarithmic scale (Figure 7b). The slope of the straight line (1.1465) revealed that the GH-Co_33_Cu_67_ catalyzed HAB followed a first order reaction with regard to the catalyst amount.

**Figure 8:**
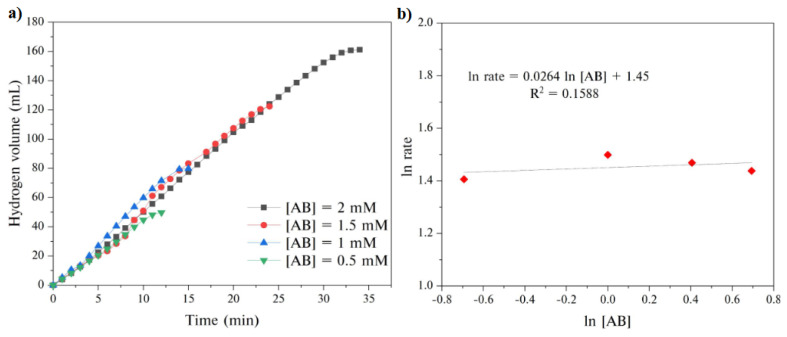
**a)** The plots of the volume of hydrogen versus time for the GH-Co_33_Cu_67_ catalyzed AB hydrolysis at different AB concentrations, **b)** the plot of ln rate versus ln [AB].

A series of experiments were also performed by using different AB concentrations whereas keeping the catalyst amount constant at 10 mg. As clearly be concluded from the plots in Figure 8a, increasing AB concentration did not have a significant effect on the hydrogen generation rate. This result was well illustrated by the slope of Figure 8b calculated by plotting the hydrogen generation rate versus AB concentration, both on a logarithmic scale. The calculated slope of 0.026 indicated that GH-Co_33_Cu_67_ catalyzed AB hydrolysis was the 0th order with regard to AB concentration. Consequently, the hydrogen generation from AB hydrolysis using the synthesized GH-Co_33_Cu_67_ as a catalyst is independent of the concentration of AB.

**Figure 9:**
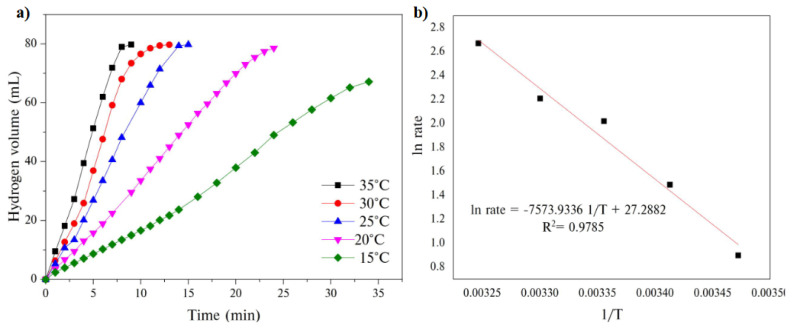
**a)** The plots of the volume of hydrogen versus time for the GH-Co_33_Cu_67_ catalyzed AB hydrolysis at different temperatures. **b)** the Arrhenius plot (lnk vs 1/T).

Finally, GH-Co_33_Cu_67_ catalyzed AB hydrolysis was also investigated at different temperatures (Figure 9a). The hydrogen generation rates noticeably increased from 319.73 to 1731.87 ml H_2_ g_catalyst_^−1^ min^−1^ with increasing the temperature. The apparent rate constants, k_app_, of the catalytic AB hydrolysis at different temperatures were calculated from the slope of the linear part of each plot in Figure 9b. Next, the plot of ln k_app_ versus 1/T was constructed to calculate the activation energy of AB hydrolysis catalyzed by GH-Co_33_Cu_67_ according to the Arrhenius equation [76]. Based on the slope of the straight line shown in Figure 9b, the activation energy was calculated as 63.16 kJ mol^−1^.

**Figure 10:**
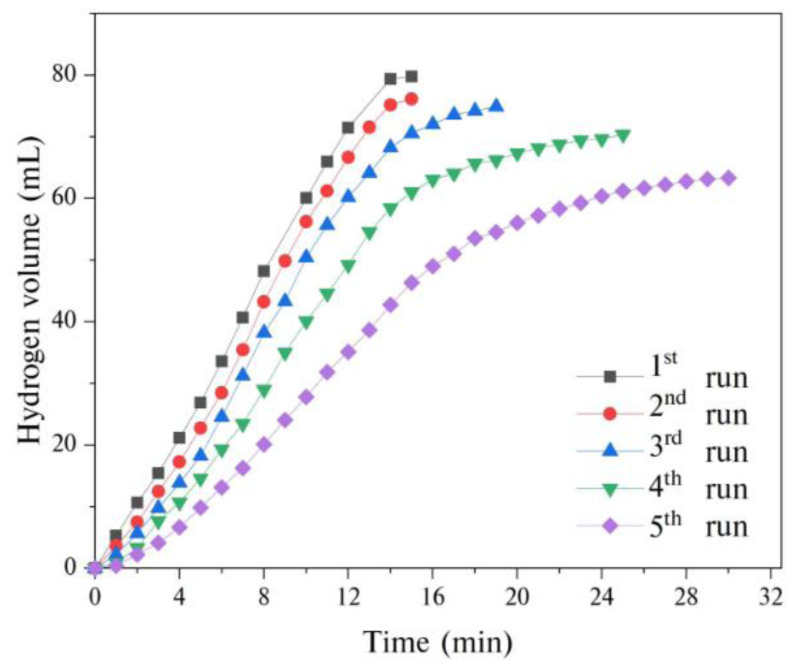
Time versus volume of hydrogen plots for the reusability of GH-Co33Cu67 nanocatalysts in the hydrolysis of AB.

The reusability of GH-Co_33_Cu_67_ nanocatalysts in the hydrolysis of AB was also investigated by performing a five-run reusability test. Figure 10, which presents the plots of hydrogen volume versus time for the reusability test, reveals that GH-Co_33_Cu_67_ nanocatalysts preserved 83.8% of its initial activity after the 2nd run, 71.9% after the 3rd run, 61.3% after the 4th run, and 45.1% after the 5th run. These results confirm that GH-CoCu nanocatalysts are not very stable catalysts in the hydrolysis of AB, but they might be promising to be fully reusable catalysts if the necessary treatments are applied after each cycle.

## 4. Conclusions

In summary, GH-CoCu nanocomposites with different Co and Cu initial loadings were synthesized by a one-pot synthesis protocol that allowed the simultaneous self-assembly of GO sheets into GH and the reduction of Co(II) and Cu(II) ions into the CoCu alloy NPs. The synthesis method was based on the combination of the hydrothermal and polyol process using EG as reducing agent and a surfactant at the same time along with the eventual effect of the GH network for a better stabilization of the generated CoCu NPs. As a result, we for the first time achieved CoCu alloy NPs anchored on GH networks using a simple and cost-effective one-pot synthesis procedure. The molar composition investigation showed that the highest hydrogen generation rate was provided by GH-Co_33_Cu_67_ nanocatalyst, which exhibited a better catalytic performance than its Co and Cu counterparts revealing the synergy generated from the formation of CoCu alloy NPs. The kinetic study performed on the as-prepared GH-Co_33_Cu_67_ nanocatalysts showed that AB hydrolysis reaction was a first order and a zero-order with respect to the catalyst and AB concentrations, respectively. The activation energy obtained was 63.16 kJ mol^−1^. The reusability test showed that the GH-CoCu anocatalysts are durable up to the 3rd consecutive runs, but their durability decreases after the 3^rd^ run. Overall, this study showed that GH supported with CoCu alloy NPs are promising new nanocomposites that can be applied for hydrogen generation from AB hydrolysis and have a great potential to be employed and investigated in various application fields.

## Figures and Tables

**Table:** Theoretical and experimental alloy compositions of as-synthesized GH-CoCu nanocatalysts.

Theoretical alloy composition based on the initial metal salt amounts	Co_50_Cu_50_	Co_50_Cu_50_ (annealed)	Co_62.5_ Cu_37.5_	Co_37.5_ Cu_62.5_
Experimental alloy composition determined by ICP-MS analysis	Co_33_Cu_67_	Co_34_Cu_66_	Co_45_Cu_55_	Co_23_Cu_77_
